# Supplementary material for: Supervised learning in spiking neural networks with FORCE training
Source: Nat Commun. 2017 Dec 20;8:2208. doi: 10.1038/s41467-017-01827-3 (PMC5738356; doi:10.1038/s41467-017-01827-3)
Supplement: Supplementary file 1 — Supplementary Information [file 41467_2017_1827_MOESM1_ESM.pdf]

Supplementary Material for Supervised Learning in Spiking Neural Networks with FORCE Training

Wilten Nicola and Claudia Clopath

October 16, 2017

All Supplementary Movie and Audio clips are in real time, with the exception of Supplementary Movie 5.

Supplementary Audio 1

A 1:40 audio clip of the short Ode to Joy spiking network reproducing the song. The decoded network output is used as the envelope for the corresponding note frequency. The wave forms used to generated the song are sinusoidal.

Supplementary Movie 1

Two consecutive replays of the songbird supervisor with the neuronal activity in the form of $\boldsymbol{r}(t)$, the synaptically filtered and normalized spike trains plotted as a block matrix on the right.

Supplementary Movie 2

Replay of the long Ode to Joy clip with the HDTS components in the bottom, and the decoded notes in the top. The network activity is shown on the right in the form of $\boldsymbol{r}(t)$. The video clip is 100 seconds long.

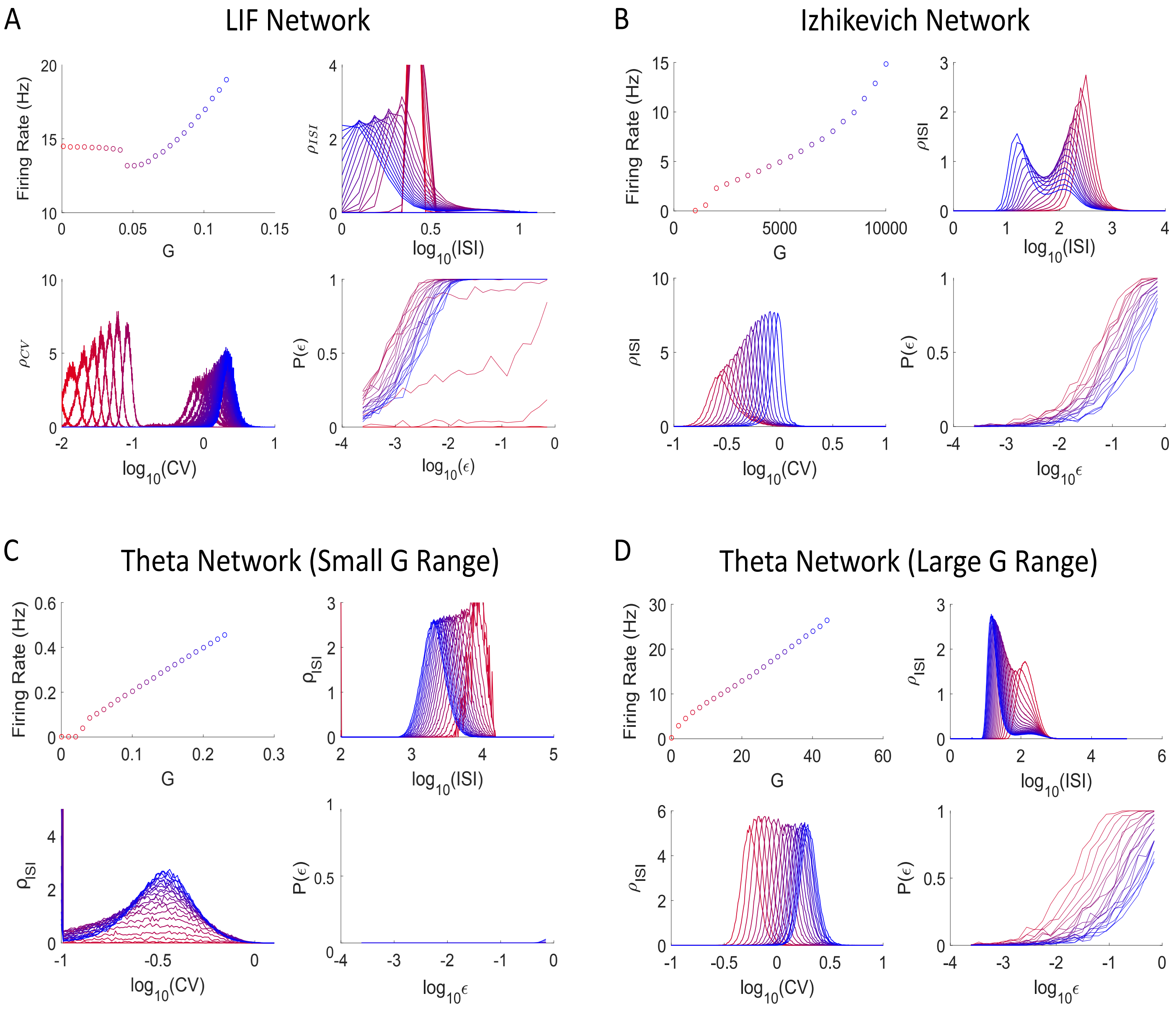

Supplementary Figure 1: The connectivity parameters G are varied over a discrete mesh to determine the spiking behavior for a network of Leaky integrate and fire (A), Izhikevich neurons (B), Theta neuron (C) and Theta neurons over a larger parameter range (D). The firing rate is shown in the top left corner as a function of G, with increasing values of G being indicated on a red-blue colour scale for each neuron model. The distribution of interspike intervals (top right) and coefficients of variation (bottom left), in addition to the probability $P(\epsilon)$ of a perturbation of size ϵ destabilizing the network is plotted (bottom right). All networks consisted of 2000 neurons, with statistics computed after an initial 5 (Izhikevich and LIF) or 10 second (Theta neuron) transient. The Izhikevich and theta neurons immediately transition to chaotic spiking from quiescence ($G \approx 0.02$, $G \approx 1000$, respectively), as their bias currents are set to the rheobase. The LIF network transitions from tonic spiking at a constant rate to chaotic spiking ($G = 0.04$). All networks exhibit a possible transition to rate chaos for high enough G, as indicated by bimodal interspike-interval distributions for sufficiently high G. The probability of divergence, $P(\epsilon)$ was measured by following a reference trajectory and perturbing off of it every 300 ms. The reference trajectory is followed after the initial transient network behavior. The N-dimensional perturbations had a magnitude of ϵ in a randomly generated, unbiased direction and were applied to the postsynaptic filters, $r_i(t)$. The perturbations off the reference trajectory were recorded and classified as either divergent or convergent back to the reference trajectory.

Neuron Model	Example	λ^{-1}	G	Q	Training Time	Firing Rate
Izhikevich Model ($\tau_D = 20$ ms)						
	5 Hz sine wave	2 ms	$5 * 10^3$	$5 * 10^3$	5 s	35.7 Hz
	5 Hz sawtooth wave	2 ms	$5 * 10^3$	$4 * 10^3$	5 s	36.8 Hz
	Van der Pol (Harmonic)	2 ms	$1 * 10^4$	$9 * 10^3$	5 s	43.4 Hz
	Van der Pol (Relaxation)	2 ms	$1 * 10^4$	$2 * 10^4$	5 s	41.9 Hz
	$sin(8\pi t)sin(12\pi t)$	2 ms	$1 * 10^4$	$9 * 10^3$	5 s	47.1 Hz
	$sin(8\pi t)sin(12\pi t) + 0.05\zeta_i$	2 ms	$1 * 10^4$	$8 * 10^3$	5 s	47.9 Hz
(Oscillator 1)	$\frac{1}{2}sin(8\pi t) + \frac{1}{6}sin(12\pi t) + \frac{1}{4}sin(28\pi t)$	2 ms	$1 * 10^4$	$1 * 10^4$	25 s	52.3 Hz
(Oscillator 2)	$sin(4\pi t)sin(6\pi t)sin(14\pi t)$	2 ms	$1 * 10^4$	$1 * 10^4$	25 s	41.6 Hz
Theta Model ($\tau_D = 50$ ms)						
	5 Hz sine wave	0.1 ms	50	$2 * 10^4$	5 s	47.53 Hz
	5 Hz sawtooth wave	0.1 ms	50	$2 * 10^4$	5 s	57.65 Hz
	Van der Pol (Harmonic)	0.02 ms	10	10^4	5 s	18.6 Hz
	Van der Pol (Relaxation)	0.02 ms	10	10^4	5 s	13.7 Hz
	$sin(8\pi t)sin(12\pi t)$	0.1 ms	50	$2 * 10^4$	50 s	47.1 Hz
	$sin(8\pi t)sin(12\pi t) + 0.05\zeta_i$	0.1 ms	50	$2 * 10^4$	50 s	47.9 Hz
Theta Model ($\tau_D = 20$ ms)						
	$sin(8\pi t)sin(12\pi t)$	0.01 ms	25	10^4	50 s	47.1 Hz
	$sin(8\pi t)sin(12\pi t) + 0.05\zeta_i$	0.01 ms	15	10^4	50 s	47.9 Hz
LIF Model ($\tau_D = 30$ ms)						
	5 Hz sine wave	0.0025 ms	0.05	10	5 s	47.53 Hz
	5 Hz sawtooth wave	0.0025 ms	0.1	20	5 s	57.65 Hz
	Van der Pol (Harmonic)	0.0025 ms	0.05	30	5 s	18.6 Hz
	Van der Pol (Relaxation)	0.0025 ms	0.05	30	5 s	21.7 Hz
	$sin(8\pi t)sin(12\pi t)$	0.0025 ms	0.1	30	25 s	38.2 Hz
	$sin(8\pi t)sin(12\pi t) + 0.5\zeta_i$	0.0025 ms	0.1	30	25 s	33.9 Hz
LIF Model ($\tau_D = 20$ ms)						
	5 Hz sine wave	0.0025 ms	0.1	30	5 s	38.7 Hz
	5 Hz sawtooth wave	0.0025 ms	0.1	30	5 s	41.9 Hz
	Van der Pol (Harmonic)	0.0025 ms	0.05	30	5 s	25.8 Hz
	Van der Pol (Relaxation)	0.0025 ms	0.05	30	5 s	22.3 Hz
	$sin(8\pi t)sin(12\pi t)$	0.0025 ms	0.1	30	25 s	34.3 Hz
	$sin(8\pi t)sin(12\pi t) + 0.05\zeta_i$	0.0025 ms	0.1	30	25 s	34.7 Hz
LIF Model ($\tau_D = 10$ ms)						
	$sin(8\pi t)sin(12\pi t)$	0.0025 ms	0.1	30	25 s	35.0 Hz
	$sin(8\pi t)sin(12\pi t) + 0.05\zeta_i$	0.0025 ms	0.1	30	25 s	35.0 Hz

Supplementary Table 1: Parameter table corresponding to figure S3. The quantity ζ_i is an additive white noise signal with mean 0 and standard deviation 1. The integration time step was taken to be 0.01 ms, 0.05 ms and 0.04 ms for the Theta, LIF, and Izhikevich neuron models, respectively. The Δ_t parameter in all cases was 0.5 ms, 2.5 ms, and 0.8 ms for the Theta, LIF, and Izhikevich models, respectively. The code to reproduce each panel can be found on the code repository modelDB [1] under accession number 190565.

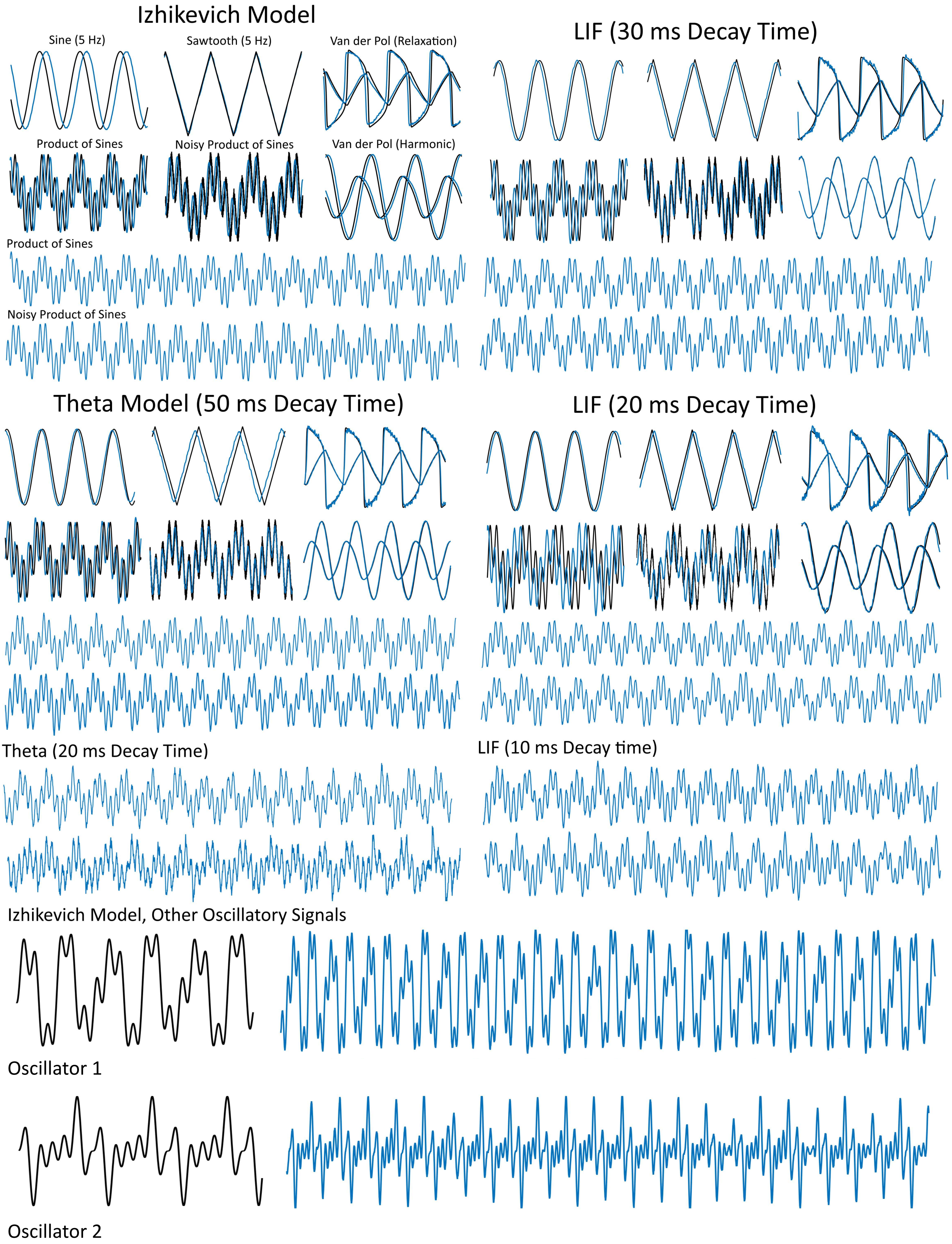

Supplementary Figure 2: FORCE Trained Oscillators for networks of theta, LIF, and Izhikevich neurons at various time constants. The parameters for each panel can be found in Table S1. The code to reproduce each panel can be found on the code repository modelDB [1] under accession number 190565.

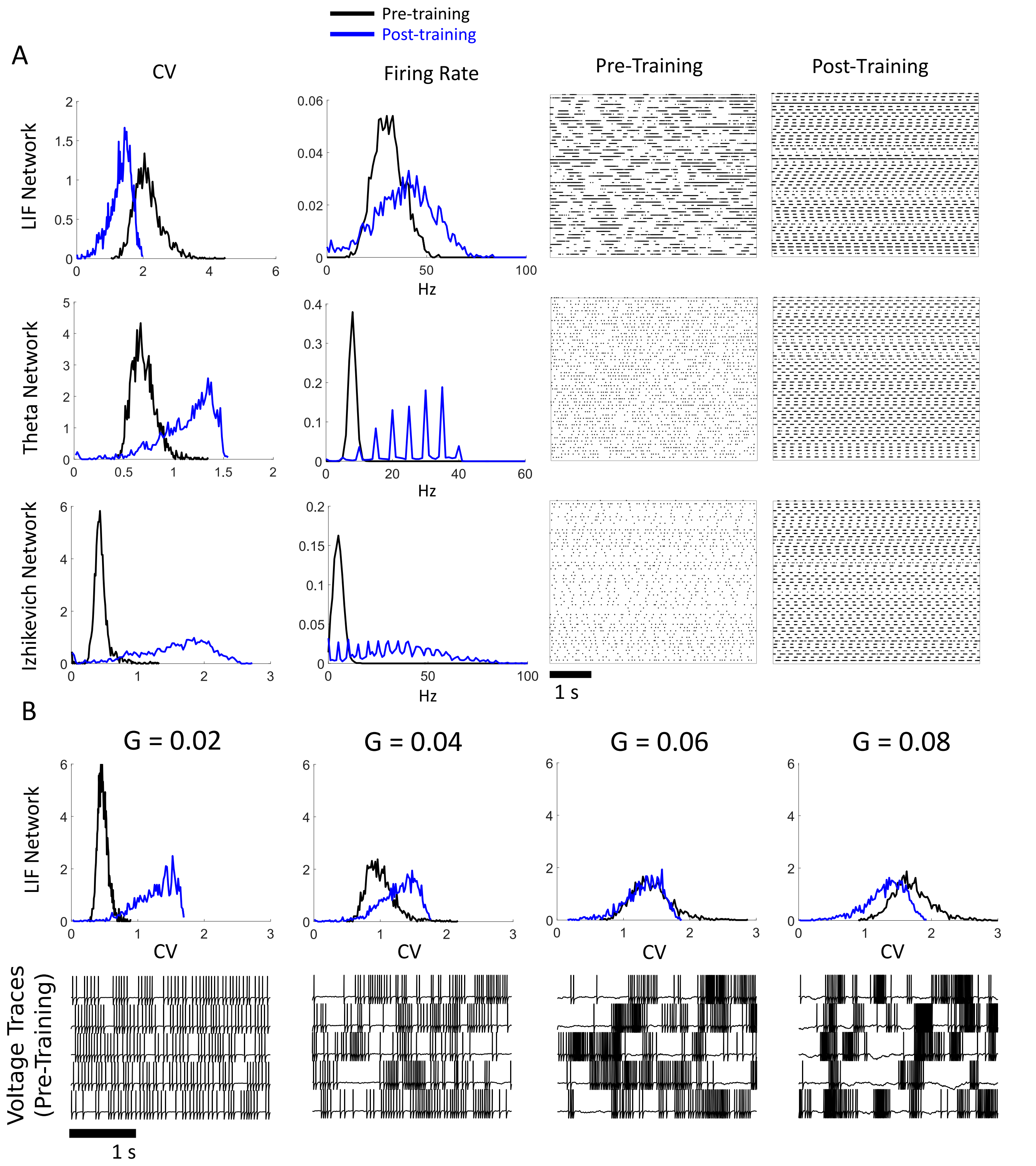

Supplementary Figure 3: (A) Networks of 2000 theta neurons (top), leaky integrate-and-fire neurons (middle), and Izhikevich models were trained to approximate a 5 Hz sinusoidal oscillator. The parameters for (A) are identical to Figure 2C in the main text. The CV distributions for these networks were centered around non-zero values for the theta and Izhikevich models. This is indicative of Poisson like random-spiking. The LIF model had a CV distribution with a mean greater than one, which corresponded to random bursting. After learning, the CV distribution increases due to the regular bursting behavior required to approximate the sinusoidal oscillation for the Theta and Izhikevich networks. If the network has strong rate fluctuations, the CV distribution decreases post-training, as in the LIF network. The average firing rates also increase after learning. (B) The LIF network is trained with the FORCE method for increasing values of G. The coefficients of variation increase with increasing G prior to training (top). The coefficients of variation increase for low G after training and decrease for large G. The voltage traces of 5 randomly selected neurons are shown for increasing G (bottom). For large G, the network has strong rate-fluctuations pre-training.

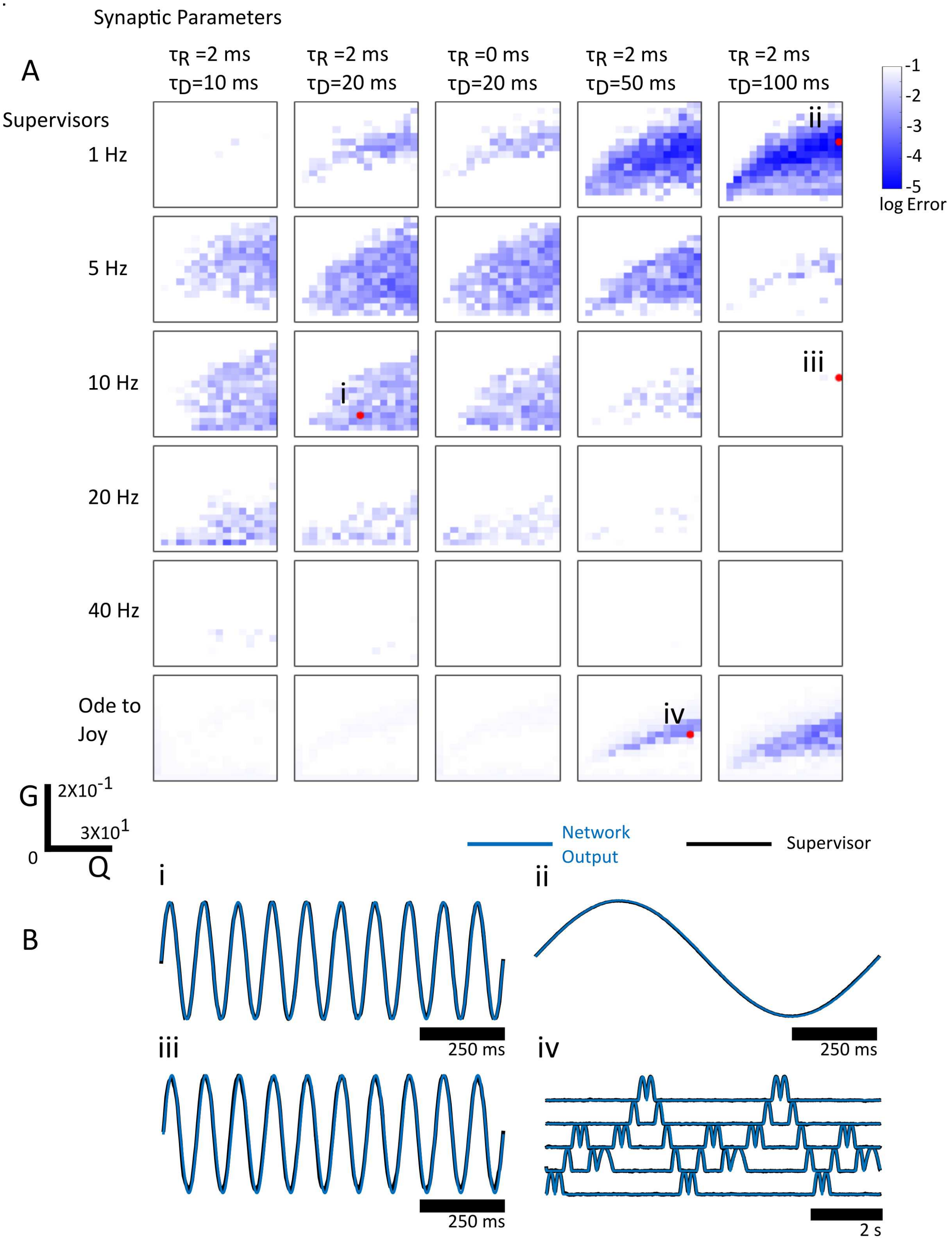

Supplementary Figure 4: (A) Networks of 2000 LIF neurons were run over 16×17 point mesh in the (G, Q) parameter space for 5 sets of sinusoidal oscillators at different frequencies, in addition to the Ode to Joy oscillator example (horizontal rows). The maximal values of (G, Q) were $(2 * 10^{-1}, 3 * 10^{1})$. The synaptic decay time constants were also varied (vertical columns). The synaptic rise time was also removed ($\tau_R = 0$ ms) for the third column. The colour indicates the magnitude of the L_2 error with darker schemes indicating greater accuracy. For the sinusoidal oscillators, 5 seconds of FORCE training with 5 seconds of testing was used while for the Ode to Joy oscillator, 85 seconds of training and 35 seconds of testing was used. Faster supervisors require faster time constants while slower supervisors require slower time constants, as indicated by the relative sizes of the convergent parameter regions. Additional parameters can be found in Table S2. (B) Four discrete simulations points are plotted in the bottom panel. These networks had the best performance in their indicated panel. The numerical value can be found in Table S2.

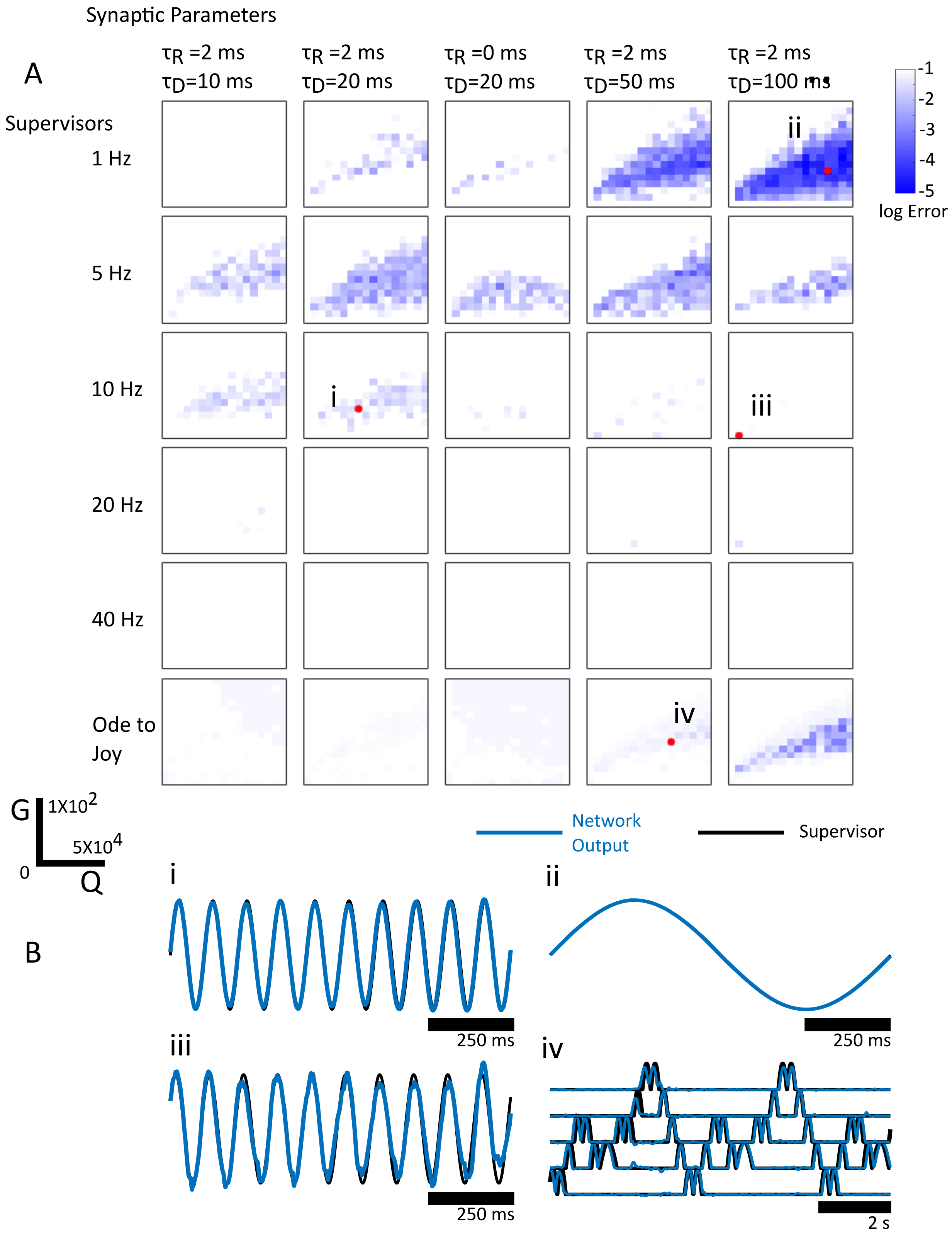

Supplementary Figure 5: (A) Networks of 2000 Theta neurons were run over 16×17 point mesh in the (G, Q) parameter space for 5 sets of sinusoidal oscillators at different frequencies, in addition to the Ode to Joy oscillator example (horizontal rows). The maximal values of (G, Q) were $(1 * 10^2, 5 * 10^4)$. The synaptic decay time constants were also varied (vertical columns). The synaptic rise time was also removed ($\tau_R = 0$ ms) for the third column. The colour indicates the magnitude of the L_2 error with darker schemes indicating greater accuracy. For the sinusoidal oscillators, 5 seconds of FORCE training with 5 seconds of testing was used while for the Ode to Joy oscillator, 85 seconds of training and 35 seconds of testing was used. Faster supervisors require faster time constants while slower supervisors require slower time constants, as indicated by the relative sizes of the convergent parameter regions. Additional parameters can be found in Table S2. (B) Four discrete simulations points are plotted in the bottom panel. These networks had the best performance in their indicated panel. The numerical value can be found in Table S2.

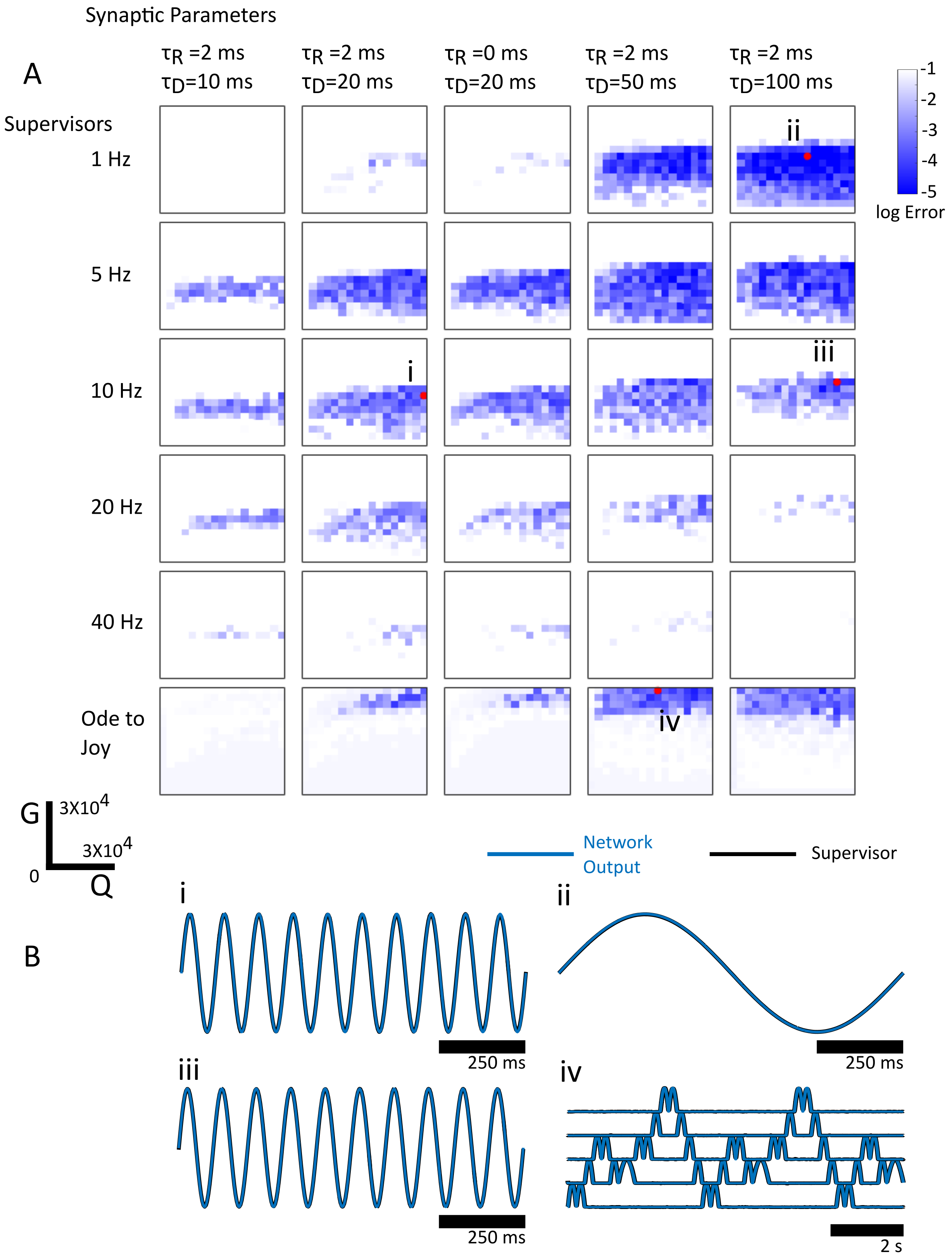

Supplementary Figure 6: (A) Networks of Izhikevich neurons were run over 16×17 point mesh in the (G, Q) parameter space for 5 sets of sinusoidal oscillators at different frequencies, in addition to the Ode to Joy oscillator example (horizontal rows). The maximal values of (G, Q) were $(3 * 10^4, 3 * 10^4)$. The synaptic decay time constants were also varied (vertical columns). The synaptic rise time was also removed ($\tau_R = 0$ ms) for the third column. The colour indicates the magnitude of the L_2 error with darker schemes indicating greater accuracy. For the sinusoidal oscillators, 5 seconds of FORCE training with 5 seconds of testing was used while for the Ode to Joy oscillator, 85 seconds of training and 35 seconds of testing was used. As in the LIF and theta models, faster supervisors require faster time constants while slower supervisors require slower time constants, as indicated by the relative sizes of the convergent parameter regions. Note that the Izhikevich model is the most robust for training as there are convergent parameter regimes that violate this trend. Additional parameters can be found in Table S2. (B) Four discrete simulations points are plotted in the bottom panel. These networks had the best performance in their indicated panel. The numerical value can be found in Table S2. Note that the Ode to Joy panels are over a smaller region in the G parameter space, from $[0, 1.5 * 10^4]$ than the sinusoidal supervisors.

Leaky Integrate and Fire Network					
RLS Parameters	$dt = 0.05$ ms, $\lambda^{-1} = 0.0025$ ms, $\Delta t = 2.5$ ms				
Time Constants (ms)	$\tau_R = 2$ $\tau_D = 10$	$\tau_R = 2$ $\tau_D = 20$	$\tau_R = 2$ $\tau_D = 20$	$\tau_R = 2$ $\tau_D = 50$	$\tau_R = 2$ $\tau_D = 100$
Sine (1 Hz)	-1.4346	-3.0605	-2.6322	-4.7398	-5.1394
Sine (5 Hz)	-2.9684	-3.6486	-3.4463	-3.7996	-2.7361
Sine (10 Hz)	-3.1256	-3.3566	-3.0229	-2.5567	-1.2777
Sine (20 Hz)	-3.6797	-2.4788	-2.2132	-1.4055	-0.8501
Sine (40 Hz)	-1.5925	-1.2403	-1.0395	-1.0609	-0.6208
Ode to Joy	-1.2165	-1.1529	-1.1869	-3.4849	-3.5075

Theta Neuron Network					
RLS Parameters	$dt = 0.1$ ms, $\lambda^{-1} = 0.01$ ms, $\Delta t = 0.5$ ms				
Time Constants (ms)	$\tau_R = 2$ $\tau_D = 10$	$\tau_R = 2$ $\tau_D = 20$	$\tau_R = 2$ $\tau_D = 20$	$\tau_R = 2$ $\tau_D = 50$	$\tau_R = 2$ $\tau_D = 100$
Sine (1 Hz)	-0.8146	-2.4401	-2.1191	-4.3523	-4.9608
Sine (5 Hz)	-2.7301	-3.3235	-3.1148	-3.5641	-3.1941
Sine (10 Hz)	-2.0599	-2.0242	-1.4525	-1.5445	-1.6085
Sine (20 Hz)	-1.3038	-0.8452	-0.4153	-1.3551	-1.4876
Sine (40 Hz)	-0.6151	-0.3796	-0.3466	-0.4114	-0.3543
Ode to Joy	-1.1527	-1.1527	-1.1527	-1.9532	-3.2607

Izhikevich Network					
RLS Parameters	$dt = 0.04$ ms, $\lambda^{-1} = 2$ ms, $\Delta t = 0.8$ ms				
Time Constants (ms)	$\tau_R = 2$ $\tau_D = 10$	$\tau_R = 2$ $\tau_D = 20$	$\tau_R = 2$ $\tau_D = 20$	$\tau_R = 2$ $\tau_D = 50$	$\tau_R = 2$ $\tau_D = 100$
Sine (1 Hz)	-0.6236	-2.9896	-2.0770	-5.3432	-6.1244
Sine (5 Hz)	-3.5554	-4.6956	-4.6090	-5.1753	-5.5939
Sine (10 Hz)	-3.7014	-4.5510	-4.1015	-4.5915	-4.7949
Sine (20 Hz)	-3.3458	-3.4526	-3.5222	-3.8267	-2.3370
Sine (40 Hz)	-2.3023	-2.3983	-2.3765	-1.5221	-1.1282
Ode to Joy	-1.1774	-4.1295	-3.6252	-4.5536	-4.5384

Supplementary Table 2: Parameter table corresponding to supplementary Figures 5-7. The quantity listed is the minimal $\log(L_2)$ run over the parameter mesh. Green colouring of a cell indicates optimality over the three neuronal models for a given time constant and supervisor. For the majority of cases, the Izhikevich had the greatest accuracy.

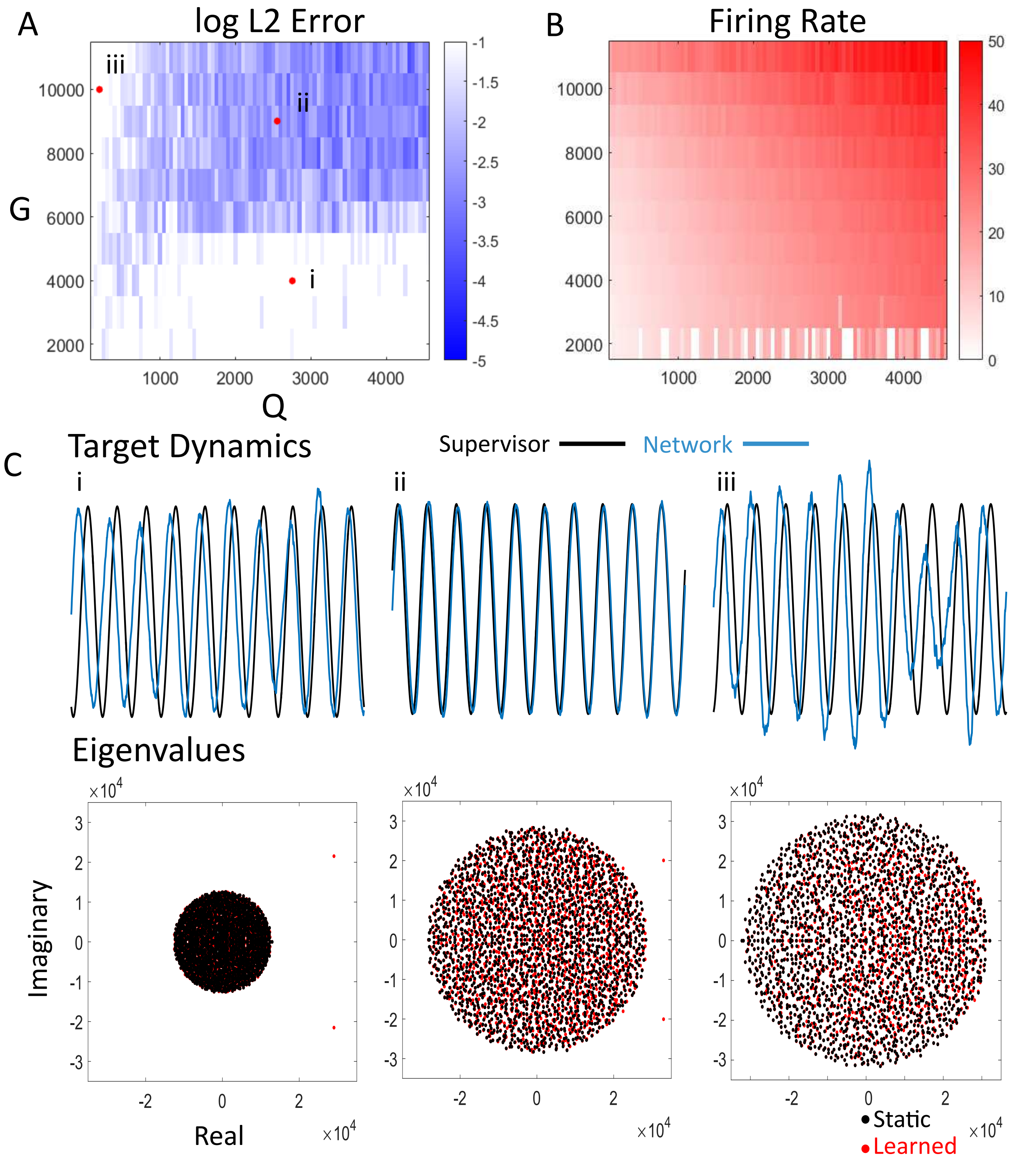

Supplementary Figure 7: (A) Networks of 2000 Izhikevich neurons were run over a 90×10 uniform mesh over the (Q, G) parameter space. Each network was tasked to learn the dynamics of a 5 Hz sinusoidal oscillation using FORCE training. 4 seconds of FORCE training were used, and the L_2 error was computed for the last 4 seconds where RLS is turned off. The colors denote the $\log(L_2)$ error with bluer colours indicating less error. The RLS parameters used here were identical to Figure 2C. (B) The average firing rate for the networks of neurons simulated over the parameter mesh in the test phase. The majority of these networks have low average firing rates (< 50 Hz). (C) The target dynamics (black) and the network approximant (blue) for three points in the mesh of simulations corresponding to networks with weight matrices heavily dominant eigenvalues (left), dominant eigenvalues (middle), and no dominant eigenvalues (right). The intermediate region has the lowest L_2 error where there are dominant eigenvalues that are near the circular cloud. Red dots denote the eigenvalues after training while black dots correspond to eigenvalues before training. Note that the transition to chaotic spiking occurs at $G \approx 10^3$ for the Izhikevich network at these parameters.

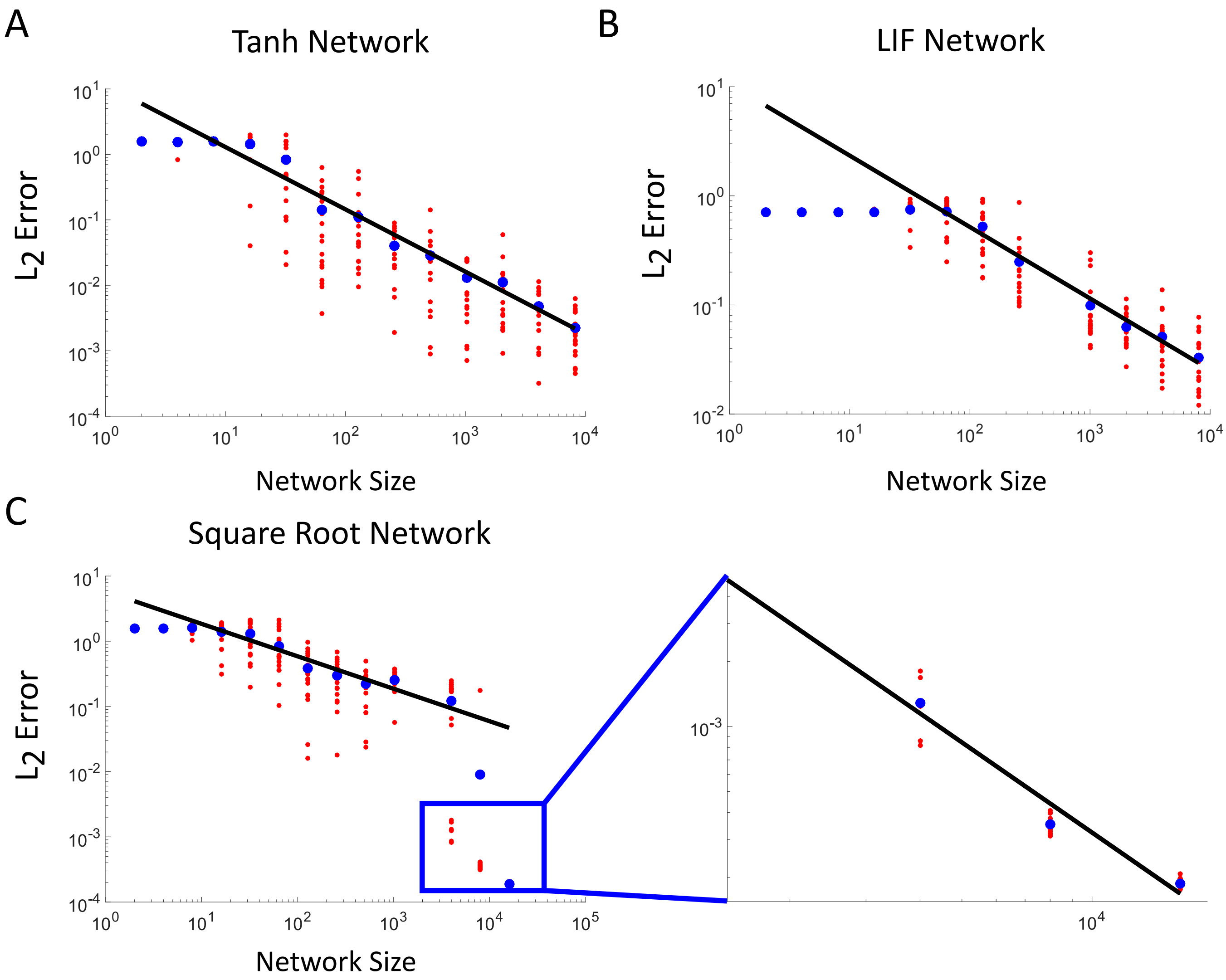

Supplementary Figure 8. Networks of rate neurons and spiking neurons were simulated for different network sizes to determine the convergence rate for FORCE training. The rate networks were either $\sqrt{x}$ (A) or $\tanh(x)$ (C) firing rates while a LIF spiking network (B) was used. The networks ranged from $O(1)$ to $O(10^4)$ with a 5 Hz sinusoidal oscillator as a supervisor and 5 seconds of FORCE training. The networks were repeatedly simulated 20 times at each network size for accurate estimates of the mean behavior. The slope of the lines of best fit to the mean $\log(L_2)$ error were -0.952 and -0.65 for the $\tanh(x)$ and LIF networks, respectively. The square root network exhibited a bimodal distribution of errors for intermediate N values. For small N values, the slope was calculated as -0.498 and stabilized to -1.385 for larger N. As $N \to \infty$, the rate networks scale faster ($\approx N^{-1}$) than the spiking network ($\approx N^{-1/2}$). The latter convergence rate is strongly indicative of a rate coding scheme.

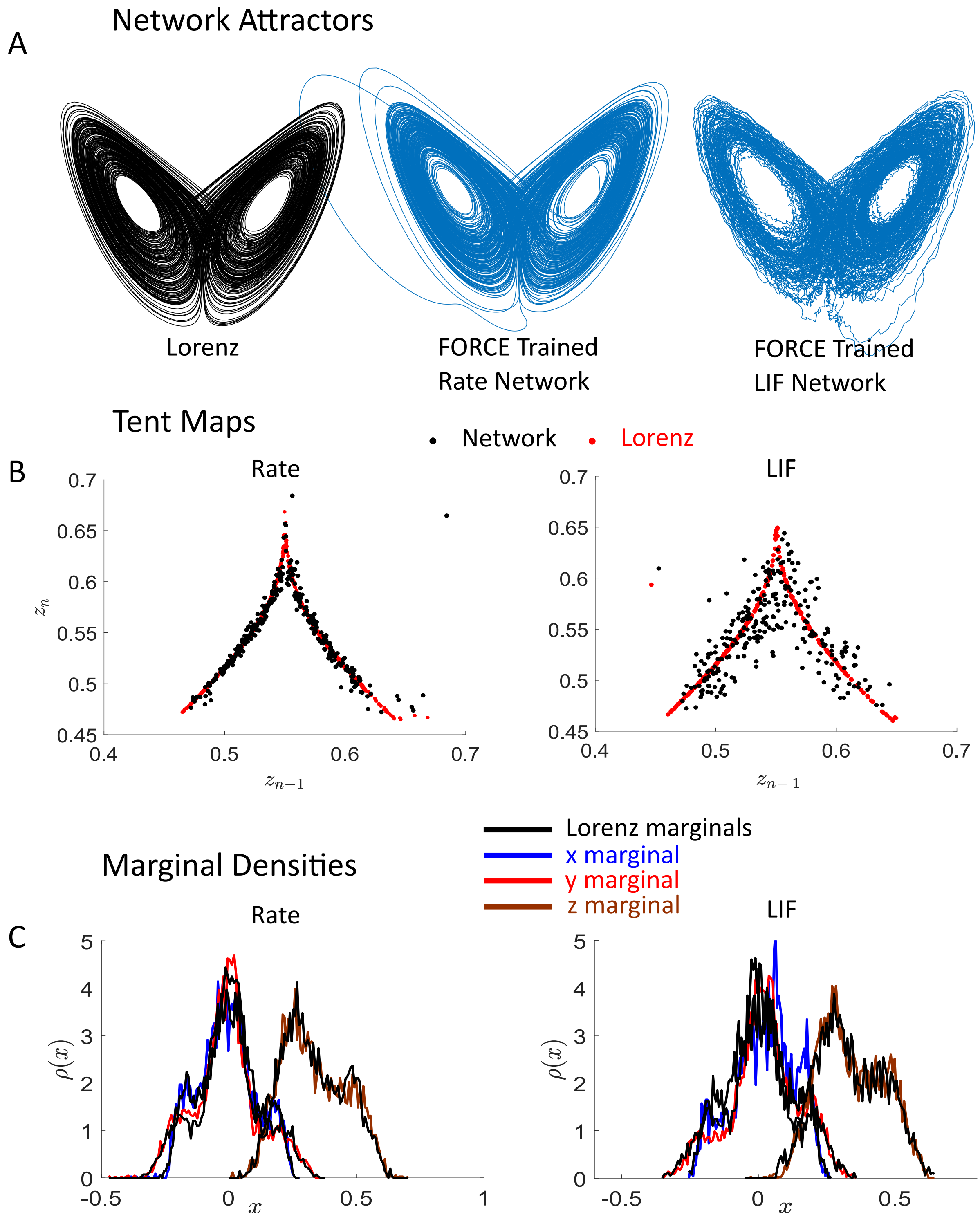

Supplementary Figure 9: Networks of $\tanh(x)$ rate neurons (left column), and networks of leaky integrate-and-fire (LIF) spiking neurons were FORCE trained on the Lorenz system in the chaotic regime. (A) Both networks can mimic the butterfly attractor (top, x vs z view). (B) The rate network (black dots) can also reproduce the stereotypical tent map, z_{n-1} vs z_n that the Lorenz system produces (red dots). The spiking network has some difficulty in producing the tent map. This is indicative of errors in the short time scale dynamics. (C) On longer time scales, both rate and spiking networks mimic the dynamics on the attractor properly as indicated by the marginal densities of the three dynamical variables. These were generated by randomly sampling 5000 points on the Lorenz system, the rate network, and the spiking network post-training. The samples were used to construct marginal density estimates in the (x, y, z) variables for comparison with the L_2 norm. The estimated Lorenz density served as the reference density. The L_2 errors were 0.27,0.30,0.24 for the rate network in the (x, y, z) variables respectively, while they were 0.52,0.38,0.30 for the spiking network. This indicates comparable levels of performance in reproducing the attractor geometry. Both rate networks and spiking networks consisted of 5000 neurons each with default parameters used in the LIF implementation (with $W = 30$, $G = 0.1$, $\lambda^{-1} = 0.0025ms$, $\Delta_t = 0.5ms$, 125 seconds of FORCE training) and the default parameters used in the tanh network (272 seconds of FORCE training, see [2] for more details and code). A Q parameter was also needed in the FORCE implementation of the tanh network ($Q = 3$) to ensure convergence.

Supplementary Note 1: The FORCE Method can Train Spiking Neural Networks to Classify Inputs

As populations of neurons encode more than just dynamical systems, we sought to determine what other potential behaviors or functions a simulated network could learn using FORCE training. For instance, human beings can naturally classify different objects as belonging to different categories or learn a particular sequence associated with the notes in a song.

To test whether a network of spiking neurons can function as a statistical classifier, we trained networks of Izhikevich neurons to classify inputs into two classes separated by either linear or nonlinear boundaries (Figure S12). The target dynamics are defined as an upward pulse when the input belongs to class 1 or a downward pulse when the input belongs to class 2. The inputs are fed in sequentially to the network via feedforward weights. Nonlinear classification in a spiking neural network is similar in complexity to the XOR task studied in [3]. Each input is followed by a short break period where the network is trained to go to a rest state. Not only can the network perform the classification task, but it can generalize. Indeed, the network learns to classify new inputs after successive presentations of training data with FORCE training, albeit with some error. In both the linear and nonlinear cases, the classification test error rate was less than 7% on test data sets.

As the majority of classifiers constructed with artificial neural networks are feedforward, we wanted to determine how the recurrent nature of the spiking network classifier influences the accuracy of the classification task. To investigate how the the network misclassifies potential inputs, we constructed peri-stimulus time histograms. The histograms were formed by repeatedly presenting the same input that were either far away from the true classification boundary or near the boundary (see Supplementary Figure 12). Unlike a feedforward neural network, the recurrent neural network used here can classify the same input as either as either class. This depends on the initial condition of the network. Thus, some of the points near the boundary are misclassified in some cases, but correctly classified at later presentations (Supplementary Figure 12). Furthermore, when the network consistently classifies points as belonging to one class or another, the variance of the voltage traces decreases across the network (Supplementary Figure 11D, Supplementary Figure 13). This result is consistent with the intracellular recordings from the primary visual cortex of cats [4, 5]. Indeed, the authors find a reduction in the voltage variance for stimuli that are both preferred and nonpreferred by neurons.

Both the linear and nonlinear classification problems were resolved with a network of 2000 Izhikevich neurons with the same synaptic and neuronal parameters as in Table 1. The remaining parameters were $G = 6 * 10^3$, $Q = 5 * 10^3$, and $\Delta t = 0.8$ ms and an integration time step of 0.04 ms. The teaching signal was constructed using the positive component of a sinusoid with a frequency of 2 Hz. The input data was uniformly distributed inside $[0, 1]^2$, with the classification boundary being $y = x$ in the linear case, and $y = \sin(2\pi x)$ in the nonlinear case. The inputs were multiplied by an $N \times 2$ input weight matrix W^{in} to yield the input current where each row of W^{in} is drawn uniformly from the circle with radius 500. The average rate for these networks was 12.5 Hz. $\lambda^{-1} = 30$ ms was used for RLS training. A total of 800 seconds of training time was used corresponding to 1600 inputs.

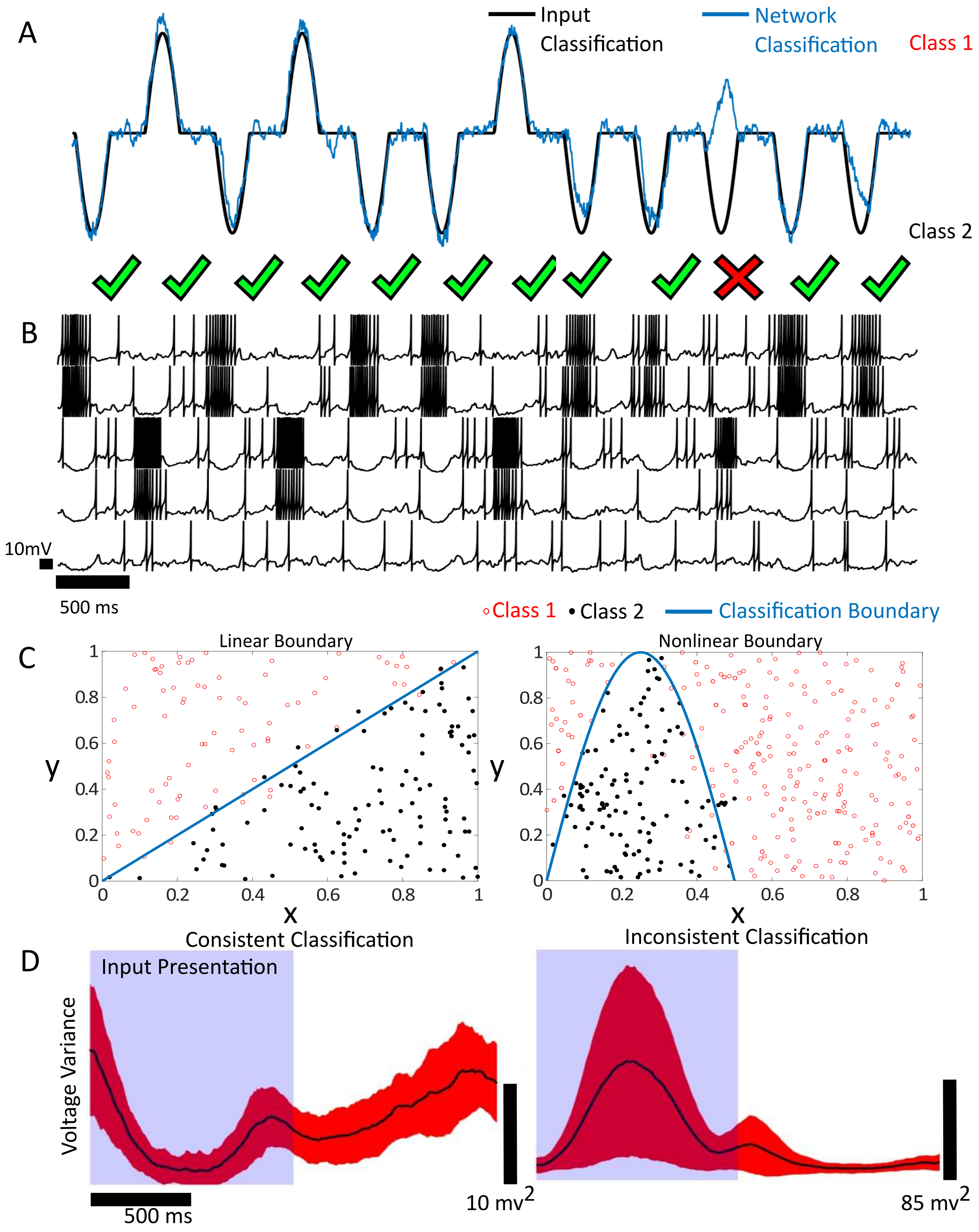

Supplementary Figure 10: (A) A binary classification task can be performed by a network of 2000 Izhikevich neurons. Positive pulses correspond to one class, and negative pulses correspond to another class. The network (blue) is trained with target data (black) using the FORCE method for 800 seconds with an input frequency of 2 Hz on a training set of inputs. The pulses are formed from the positive and negative components of a 2 Hz sinusoidal function (B) The voltage trace for 5 randomly selected neurons in the network at an identical time to (A). (C) The network correctly classifies data with linear (left) and nonlinear (right) boundaries. The network classifies points as class 1 (open red circle) or class 2 (closed black circle). The x and y axes correspond to the two dimensional input vector. The linear classification task has an accuracy of 0.93 (training time of 800 seconds, 1600 inputs) while the nonlinear task has an accuracy of 0.94 (training time of 800 seconds, 1600 inputs) on a pair of test data sets. (D) The network average voltage variance is (black, standard deviation bars in red) computed for repeated presentations of a point away from the boundary that can be consistently classified (left) and a point near the boundary that is inconsistently classified for the linear test data (right). The voltage variance only decreases for inputs that the network can consistently classify.

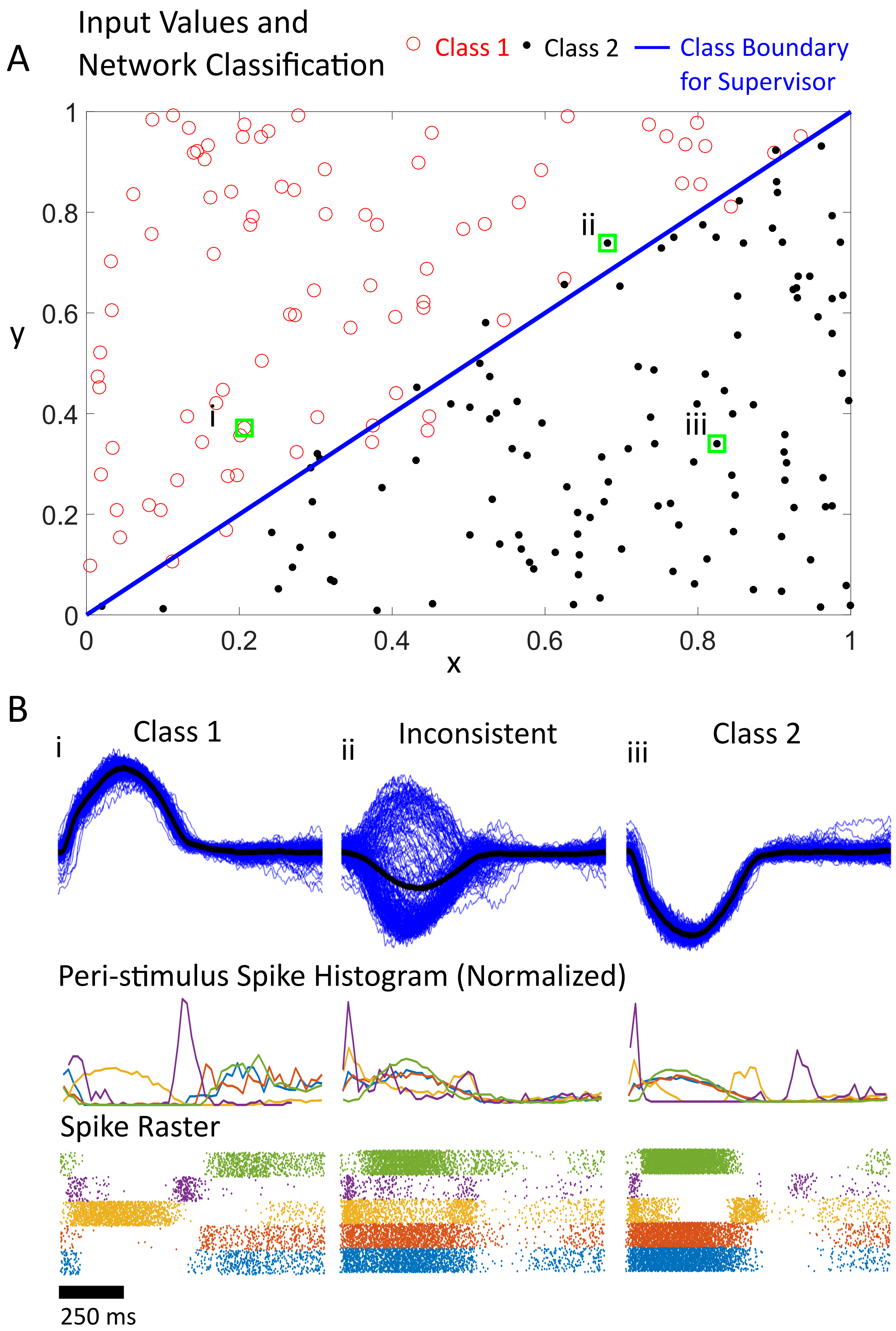

Supplementary Figure 11: (A) The inputs are classified into binary categories by the FORCE trained network. Upward pulses correspond to one class (open red circles) while downward pulses correspond to another class (closed black circles). The boundary between classes is given by $y = x$. (B) The three inputs corresponding to the three points from figure (A) are repeatedly displayed for the network to classify 50 times. A peri-stimulus time histogram is generated for the same 5-neurons in each three cases using the raster plots. The network misclassifies points near the boundary (blue line) by displaying an incorrect pulse. The misclassification depends on the initial condition of the network. Points closer to the boundary are more likely to be misclassified.

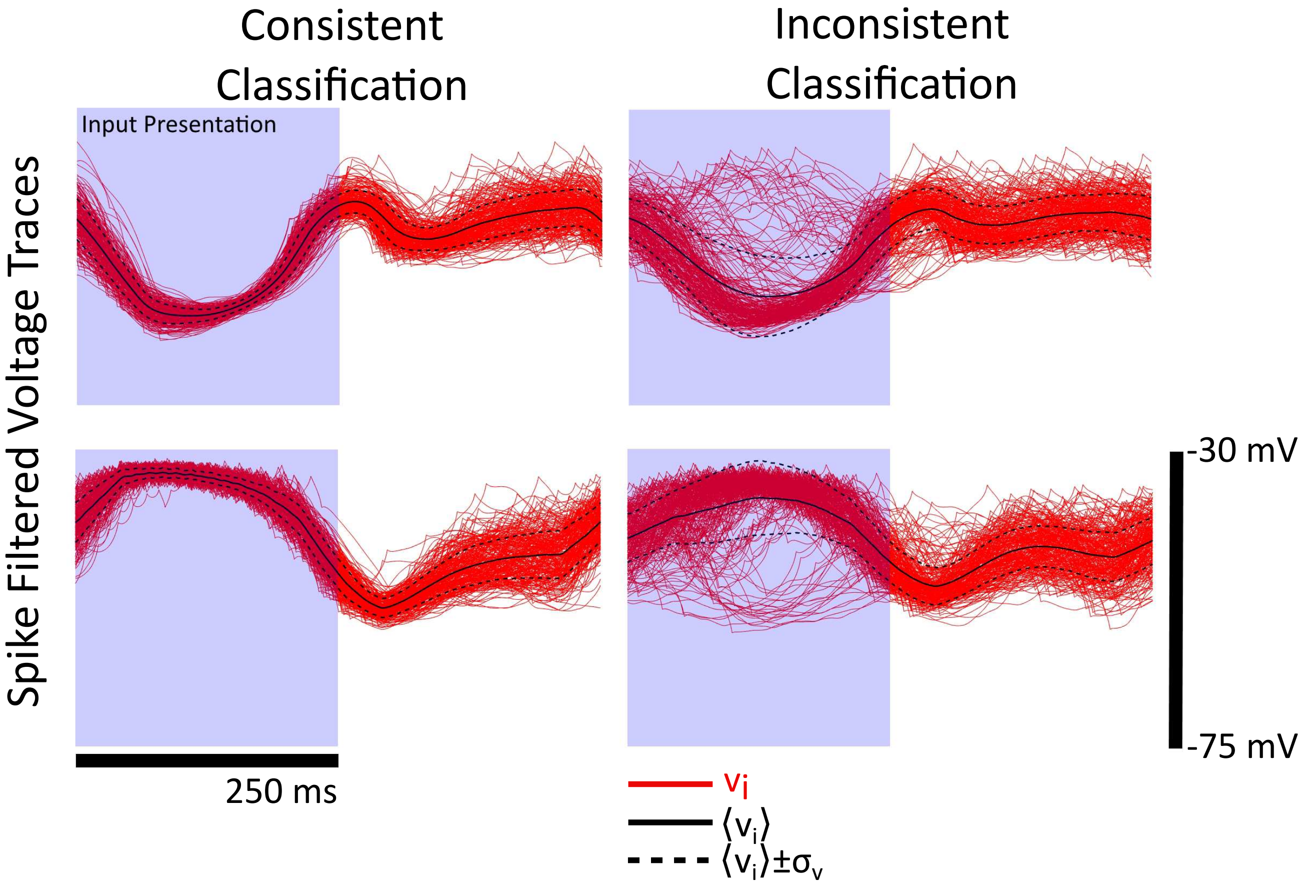

Supplementary Figure 12: The network of neurons is presented with an identical input 250 times periodically that is either consistently classified (far from the classification boundary, left column) or is inconsistently classified (near the classification boundary, right column). The voltage for a pair of neurons is box filtered with a filter of 80 ms to remove spikes. During input presentation, the voltage variance decreases regardless of whether or not the mean voltage increases (bottom) or decreases (top) for consistent classifications while the voltage variance increases with input presentation when the network inconsistently classifies a point.

Supplementary Note 2: FORCE Trained Weight Matrices that Respect Dales Law with Synaptic Boundaries

One potential solution to generate FORCE trained weight matrices that respect Dales law is through synaptic boundaries. Consider a network of N neurons with the first N_E excitatory neurons and $N_I = N - N_E$ inhibitory neurons. We start with static weight matrices ω_{ij}^0 given by the following:

$$\omega_{ij}^0 = \begin{cases} \frac{G}{\sqrt{N}p} & \text{with probability } p \text{ if } i \leq N_E \\ -\frac{G\kappa_i}{\sqrt{N}p} & \text{with probability } p \text{ if } i > N_E \\ 0 & \text{otherwise} \end{cases} \tag{1}$$

where κ_i is a term used to set $\sum_{j=1}^{N} \omega_{ij}^0 = 0$ to initialize chaotic spiking in the reservoir. These static weight matrices immediately respect Dales law. It is sufficient (but not necessary) for the low rank RLS trained weight matrix to also respect Dales law independently. This can be achieved by setting boundaries on synaptic weights when they do not respect Dales law:

$$\omega_{ij} = \begin{cases} Q\boldsymbol{\eta}_i\boldsymbol{\phi}_j & \text{if} \quad 0 \leq \boldsymbol{\eta}_i\boldsymbol{\phi}_j, \quad i \leq N_E \\ Q\boldsymbol{\eta}_i\boldsymbol{\phi}_j & \text{if} \quad 0 \geq \boldsymbol{\eta}_i\boldsymbol{\phi}_j, \quad i > N_E \\ 0 & \text{otherwise} \end{cases} \tag{2}$$

This is done dynamically as the decoders $\boldsymbol{\phi}_j$ are determined through RLS. In this way, the decoders and the approximant are determined in precisely the same way as before. The only difference is that the approximant interacts with the reservoir in a manner which obeys Dales law. The results of this procedure are shown in Figure S4 for a network of 2000 Izhikevich neurons with identical parameters to Figure 2, learning a 5 Hz sinusoidal oscillator.

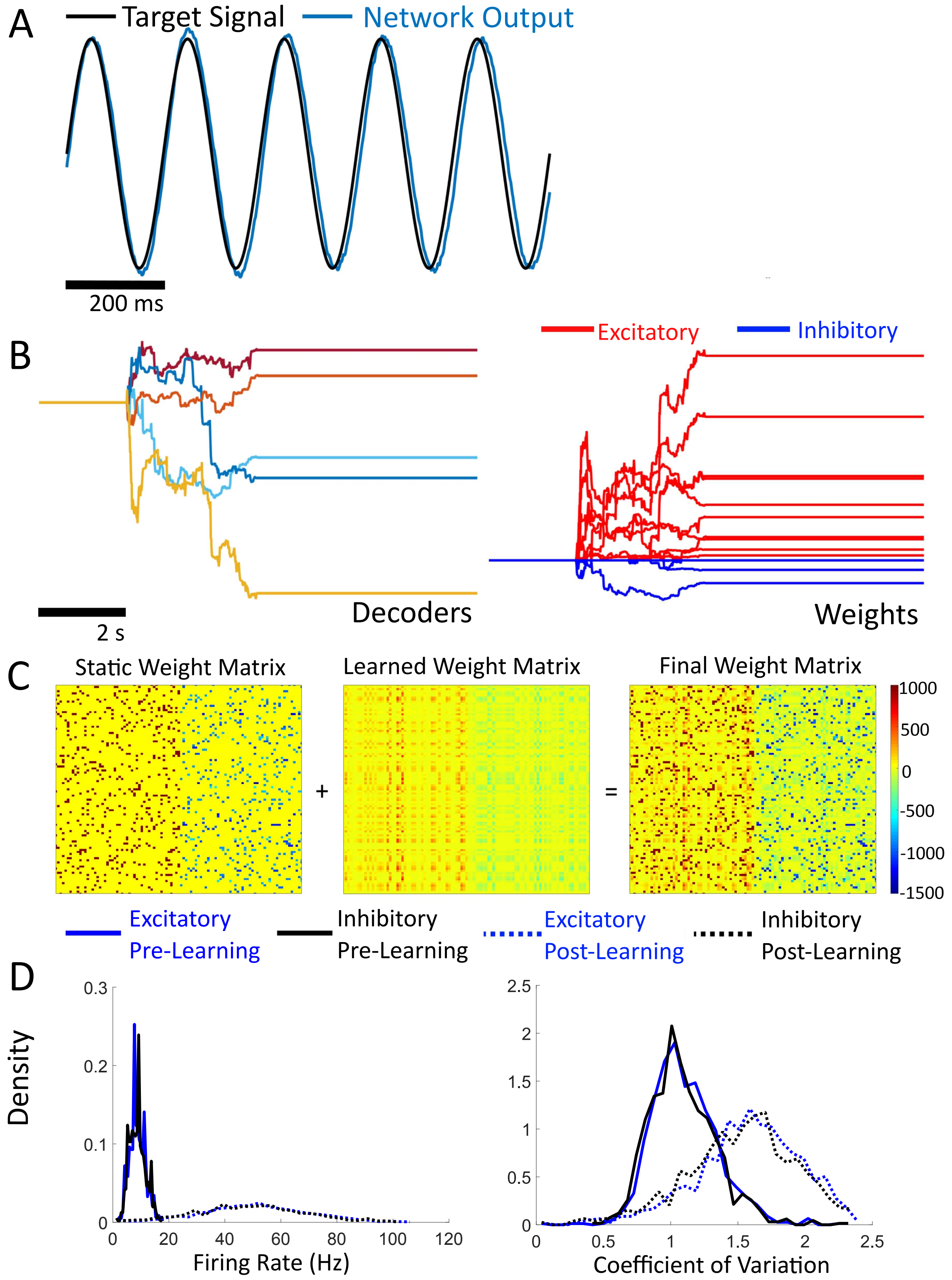

Supplementary Figure 13: (A) A network of 2000 Izhikevich neurons is trained to mimic a 5 Hz sinusoidal oscillation with weights that respect Dale's law. The first 1000 neurons are excitatory while the next 1000 are inhibitory. The weight matrices are determined such that both the static weight matrix and the learned weight matrices independently respect Dale's law. (B) The decoders for the network are learned with 3 seconds of FORCE training after a 2 second transient, and are not constrained by signs. (C) The time varying component of the weight matrix is given by $\omega_{ij} = Q\boldsymbol{\eta}_i\boldsymbol{\phi}_j$ when the weight respects Dale's law, and 0 otherwise. (D) The static weight matrix forms a backbone of strong connections that respect Dales law. The learned weight matrix forms a set of weaker connection weights used to stabilize the intended dynamics. The final weight matrix is the sum of these two and also respects Dale's law. The resulting matrix had a 0.45 degree of sparseness. (E) The spiking statistics for both excitatory and inhibitory neurons are largely identical to prior work with weight matrices that did not respect Dales law. The spiking prior to FORCE learning is near Poissonian with a coefficient of variation distribution that centers near 1 and increases post-training. The firing rates also increase post-training with an average of 50 Hz for both populations.

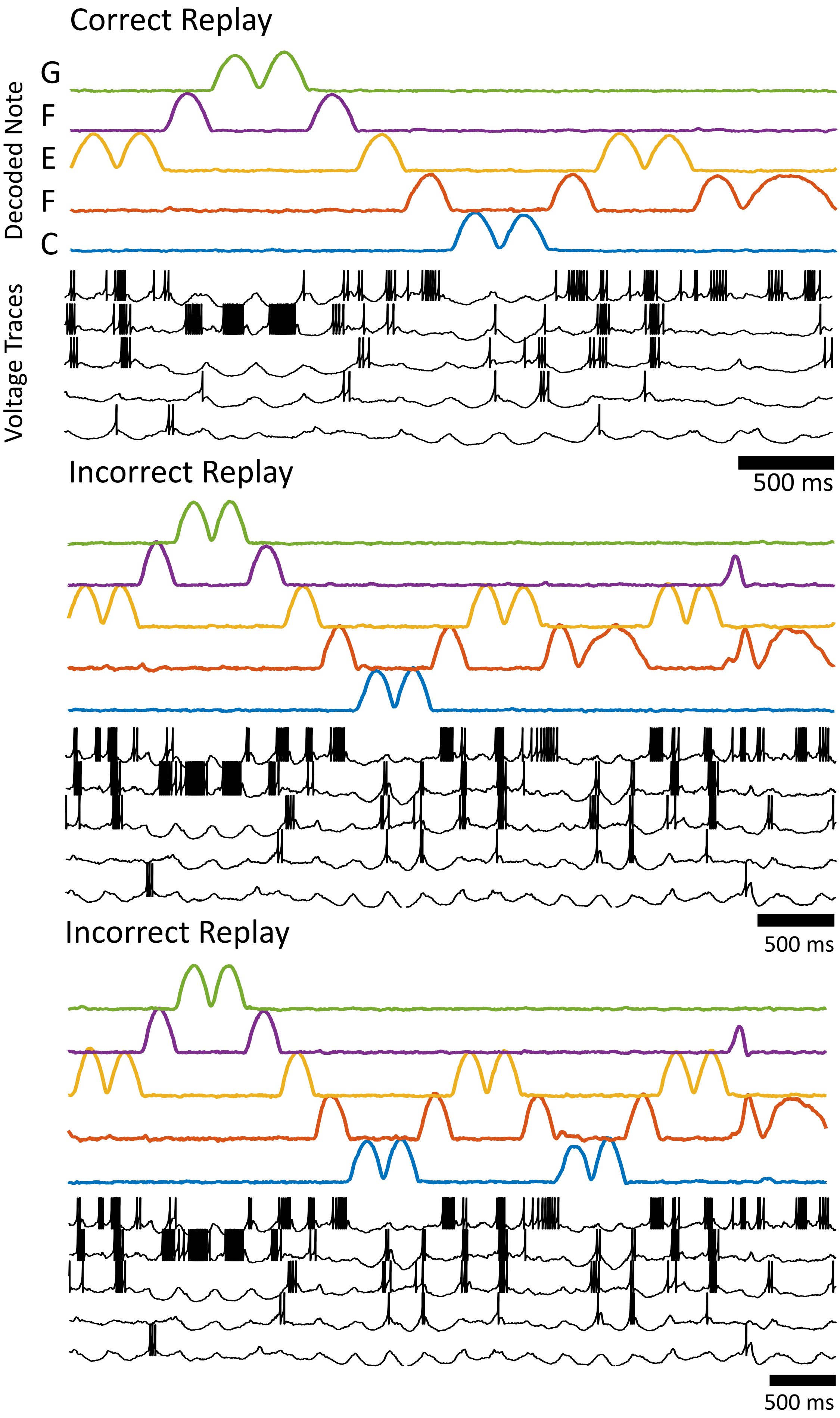

Supplementary Figure 14: A pair of incorrect repetitions of Ode to Joy after RLS is turned off (middle, bottom) in addition to a correct repetition (top). The corresponding voltage traces for 5 randomly selected neurons are shown. In both cases, the network fails to elicit the correct note in the repetition, and instead replays an alternate part of the song. The note prior to the mistake heavily influences which part of the song is replayed. In the middle replay, the error is due to the EE repeat, while in the bottom replay, the error is due to the ED repeat

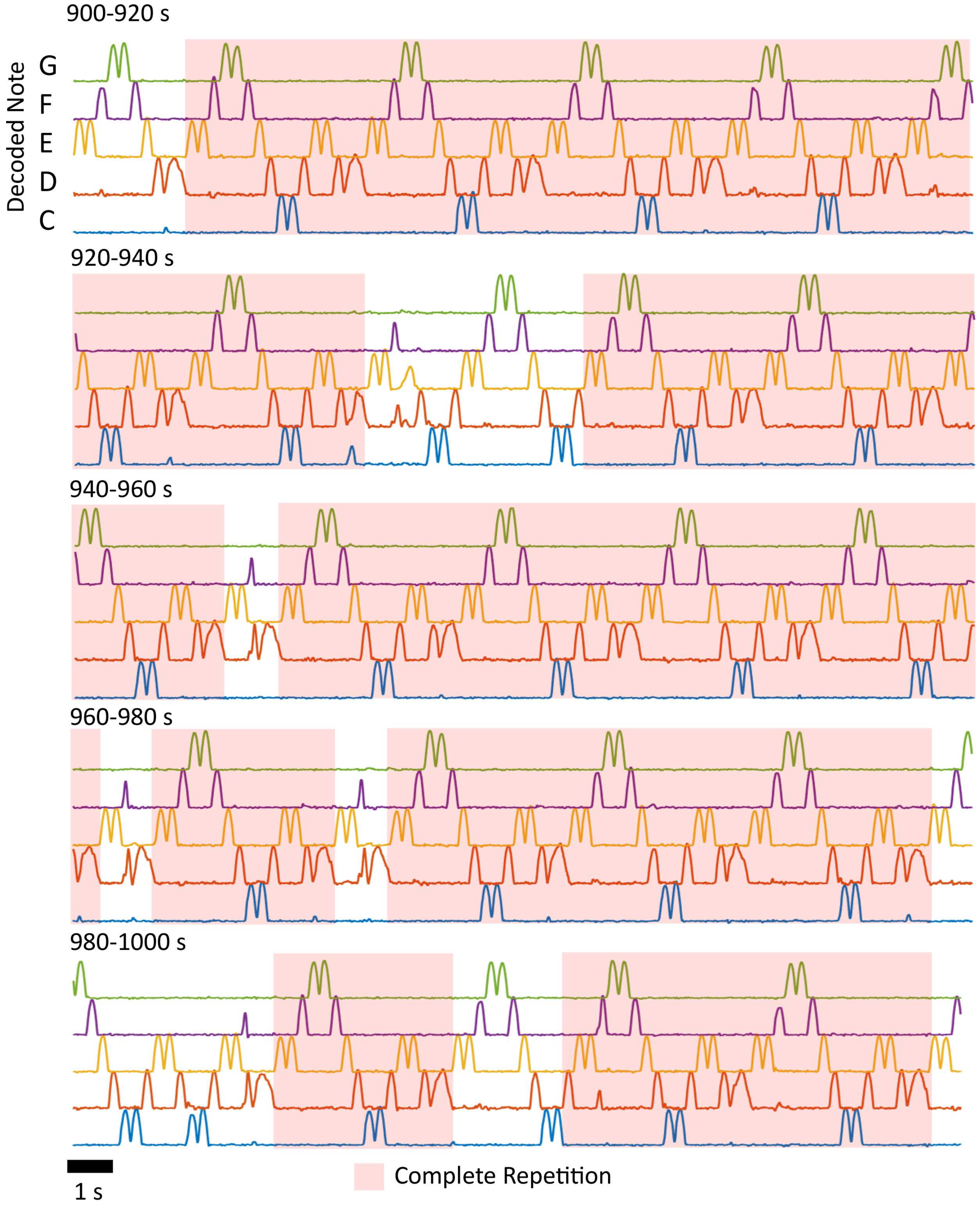

Supplementary Figure 15: Shown above is 100 seconds of the network output after RLS learning is turned off for the song example. Complete repetitions are highlighted in red.

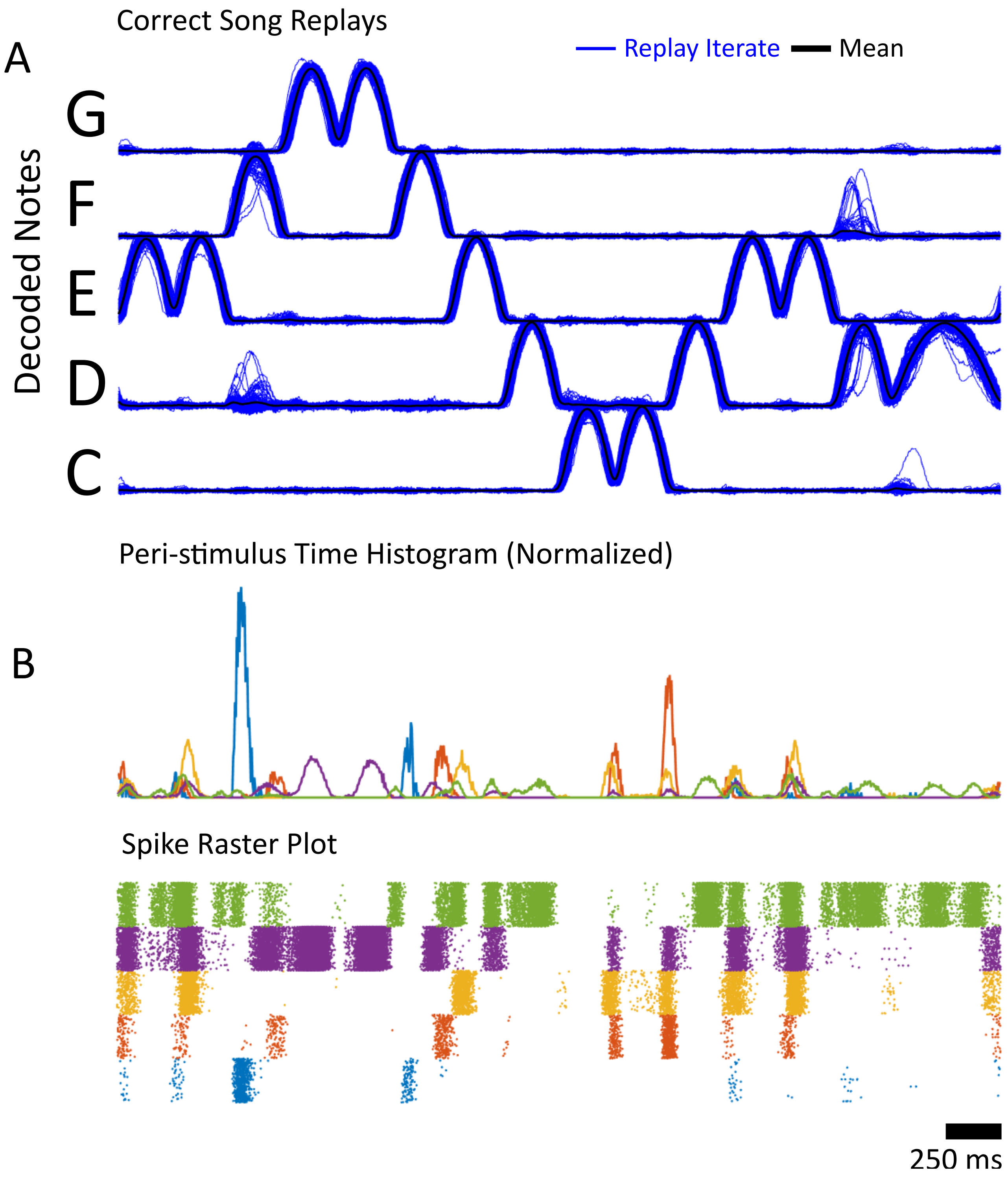

Supplementary Figure 16: (A) The network continuously replays the song for 1000 seconds after FORCE training is turned off. Correct replays are automatically classified using the L_2 error with the teaching signal and 205 correct repetitions (shown in blue) are found in 1000 seconds. This corresponds to 82% of the decoded signal. The mean of these repetitions is shown in black. (B) The peri-stimulus time histogram is constructed from the raster plots of 5 neurons. As shown in the raster plot, there is considerable trial-to-trial variability even for repetitions automatically classified as correct.

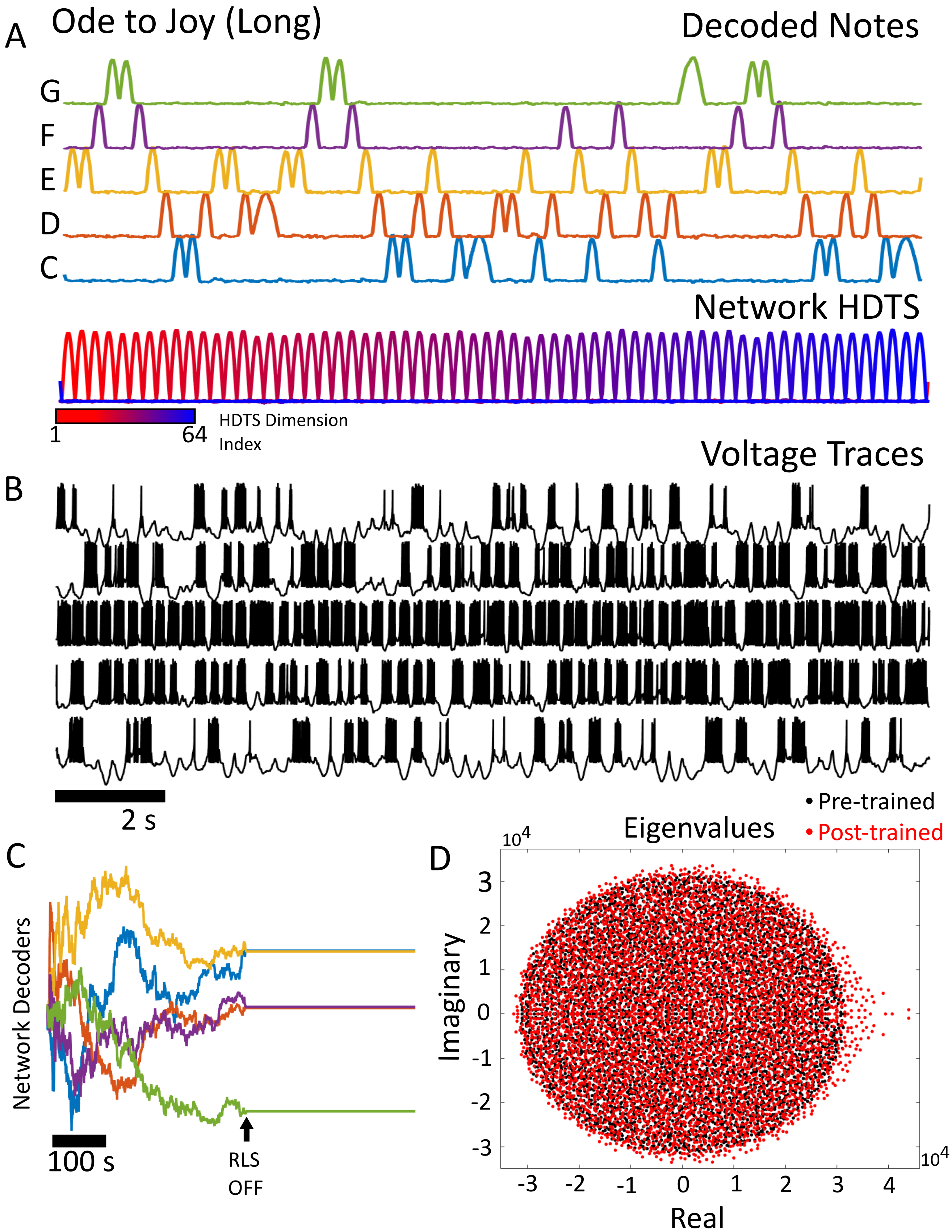

Supplementary Figure 17: (A) A network of 5000 Izhikevich neurons correctly learns the 64 note sequence to the song Ode to Joy in addition to a 64 dimensional HDTS. The HDTS corresponds to the 6th-69th components of the supervisor presented to the network. The red blue colour spectrum indicates the position of the HDTS pulse in the sequence (B) The voltage traces for 5 neurons are shown. Unlike the previous implementation we considered, the neurons in this network encode information about time through the final 64 components (the HDTS) and note through the first 5 components of the encoders and decoders. (C) The network was trained with FORCE for a period of 379 seconds, after which RLS was deactivated. The network performed the correct 64 note sequence for the remaining 321 seconds of the simulation. Unlike our previous implementation, replay of the entire song was flawless for the remainder of the simulation. (D) The eigenvalue spectrum of the weight matrix after learning shows a cloud of eigenvalues (red) near the static weight matrices original circle of eigenvalues (black) after learning this high dimensional supervisor. Note that the transition to chaos for the Izhikevich model occurs at $G \approx 10^3$

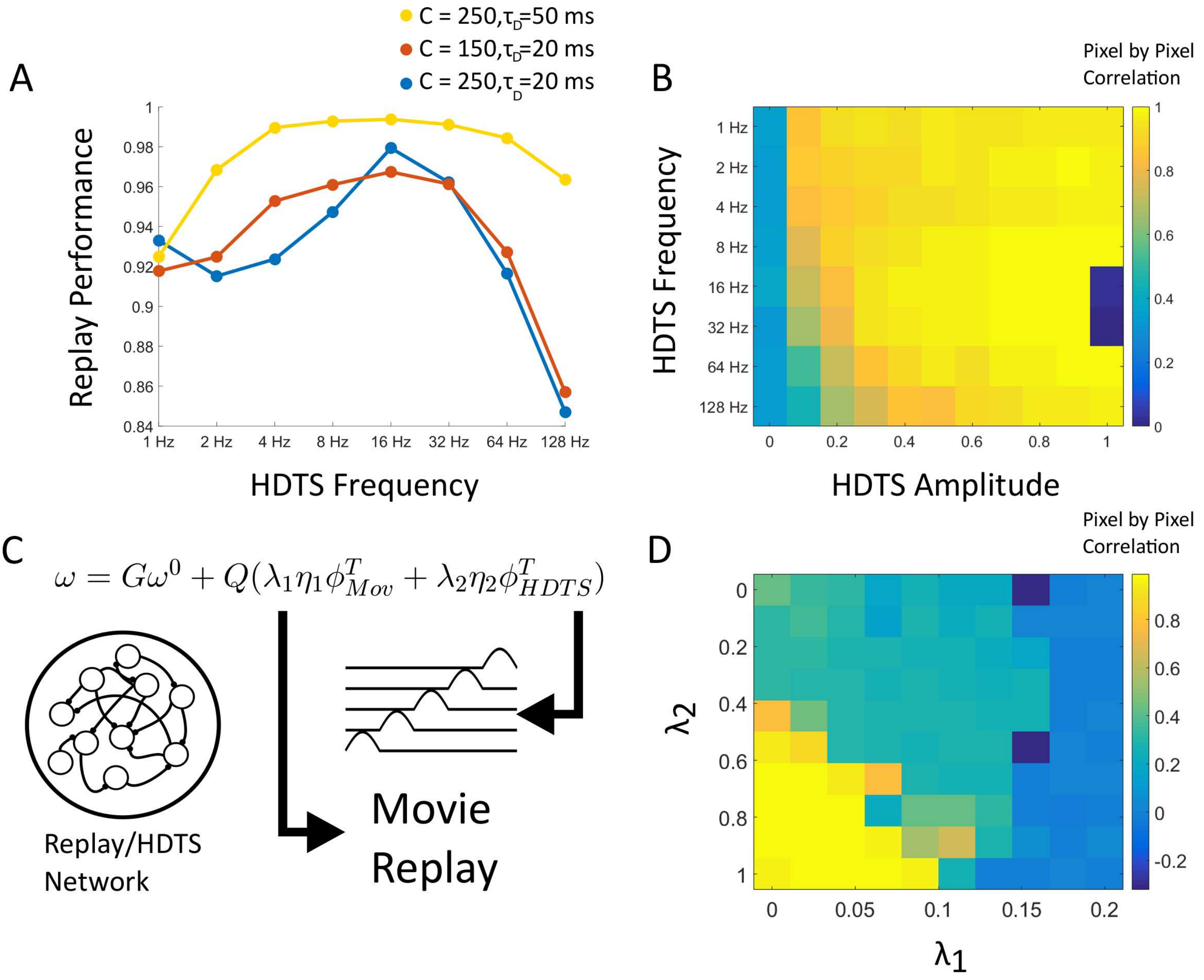

Supplementary Figure 18: The HDTS was used to train a network of 1000 Izhikevich neurons to replay the movie scene with different HDTS frequencies and HDTS input amplitudes over a 10 by 10 mesh over the parameter space. The training consisted of 74 seconds of FORCE training followed by 45 seconds of testing. (A) The HDTS amplitudes were fixed at 0.4 for three Izhikevich model parameter sets ($C = 250$, $\tau_D = 20$ ms, $C = 150$, $\tau_D = 20$, $C = 250$, $\tau_D = 50$ ms). There is a clear optimum in the 8-16 Hz range. (B) The simulated mesh for different HDTS amplitudes and frequencies for $C = 250$, $\tau_D = 20$ ms. The performance is robust for different parameters, but optimal in the 8-16 Hz range, depending on the HDTS amplitude. Similar results were found with the other parameter sets, however the $\tau_D = 50$ ms parameter set also contained global optima at $16 - 32$ Hz, depending on the frequency. (C) A network can be trained to simultaneously create its own internal HDTS and replay the movie scene. Both sets of weights (for the HDTS and the movie replay) are stored recurrently with a weighting parameter. The amount of recurrenece in the movie or in the HDTS is controlled by the λ_1 and λ_2 parameters, respectively. The networks were 2000 Izhikevich neurons with the default parameter sets (see Table 1). (D) The optimal region of convergence is in the low movie amplitude, high HDTS amplitude. Note that the movie consists of 1920 dimensions while the HDTS consists of a 64 dimensions, hence the difference in magnitude of the weighting parameters. The (G, Q) parameters for (A)-(B) and (C)-(D) were $(5 * 10^3, 4 * 10^3)$ and $(6 * 10^3, 6 * 10^3)$, respectively. Sinusoidal HDTS' were used for (A)-(B) while Gaussian signals were used for (C)-(D). All other RLS parameters were the same as in Figure 5 for both implementations.

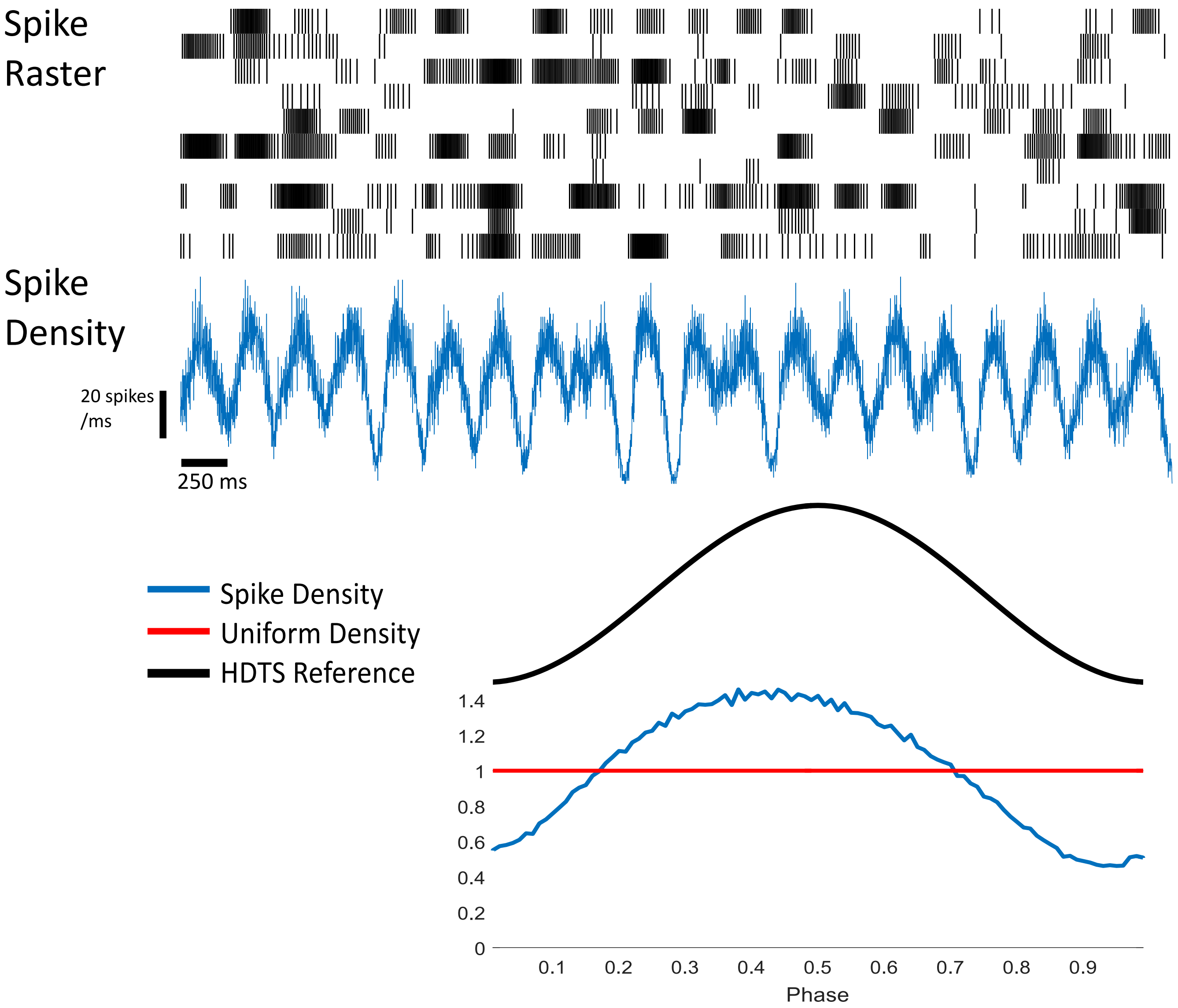

Supplementary Figure 19: The distribution of spikes is computed as a function of the phase of the HDTS input. The distribution is computed during 3 repetitions of movie replay (shown in top, 1 replay segment). The distribution is unimodal, strongly non-uniform, with a peak off the center of the input phase. This is qualitatively similar to empirically measured spike-phase distributions such as in [6]. This is the externally generated HDTS case.

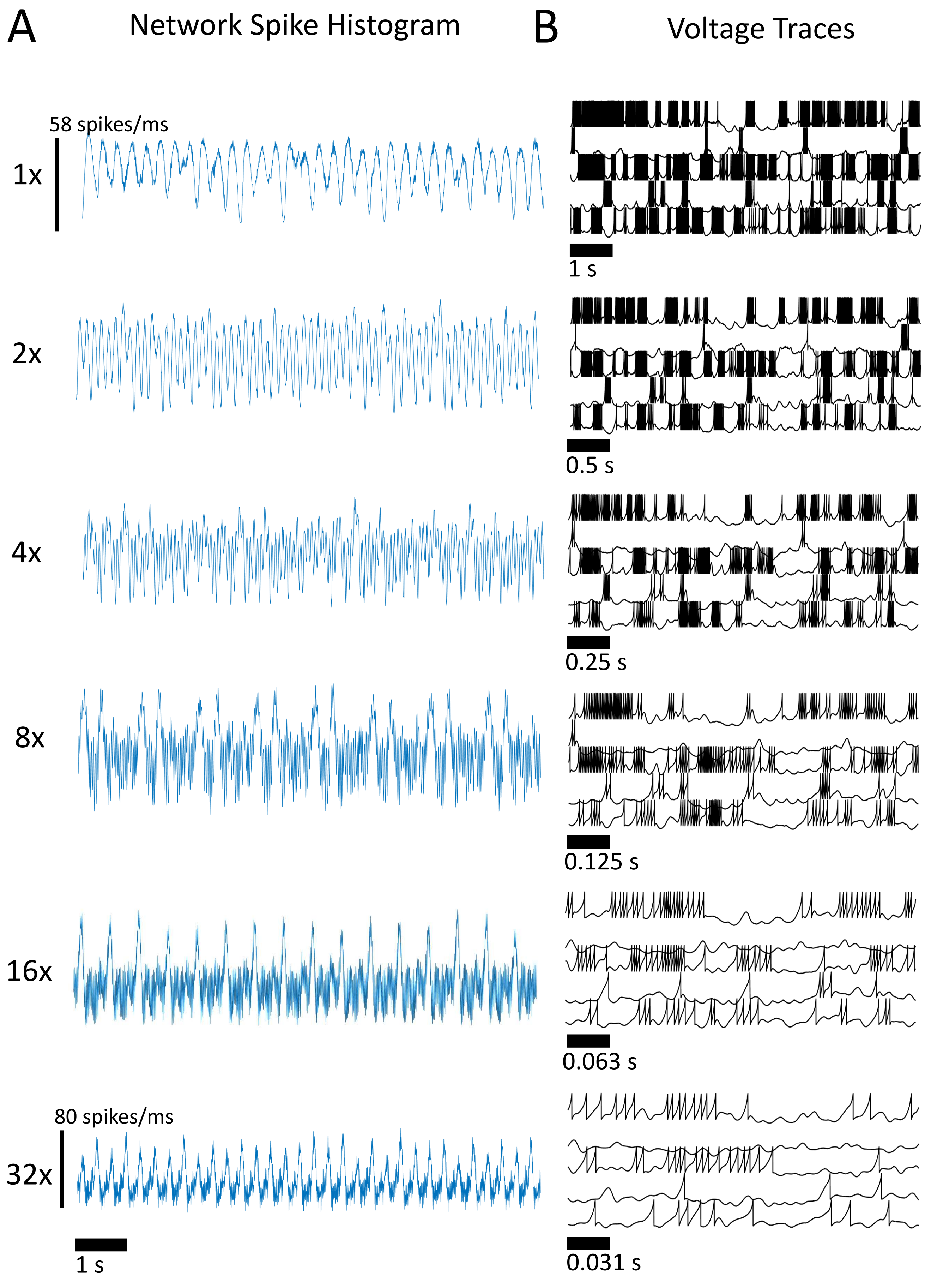

Supplementary Figure 20: (A) The spike-time histogram for the entire network is computed for different levels of HDTS compression for the external HDTS case. As the compression factor increases, the histogram has higher frequency components. The histogram is computed with 5 ms time bins. (B) The voltage traces for 5 randomly selected neurons for a single replay of the movie scene at different compression ratios. The traces are compressed by a similar ratio as the mean population activity, and the movie replay, however the relative firing rate decreases with increasing compression.

References

1. Hines, M. L., Morse, T., Migliore, M., Carnevale, N. T. & Shepherd, G. M. ModelDB: a database to support computational neuroscience. *Journal of computational neuroscience* **17,** 7–11 (2004).
2. Sussillo, D. & Abbott, L. F. Generating coherent patterns of activity from chaotic neural networks. *Neuron* **63,** 544–557 (2009).
3. DePasquale, B., Churchland, M. M. & Abbott, L. Using firing-rate dynamics to train recurrent networks of spiking model neurons. *arXiv preprint arXiv:1601.07620* (2016).
4. Churchland, M. M. *et al.* Stimulus onset quenches neural variability: a widespread cortical phenomenon. *Nature neuroscience* **13,** 369–378 (2010).
5. Finn, I. M., Priebe, N. J. & Ferster, D. The emergence of contrast-invariant orientation tuning in simple cells of cat visual cortex. *Neuron* **54,** 137–152 (2007).
6. Mizuseki, K., Sirota, A., Pastalkova, E. & Buzsáki, G. Theta oscillations provide temporal windows for local circuit computation in the entorhinal-hippocampal loop. *Neuron* **64,** 267–280 (2009).
