# Supplementary material for: Supervised learning in spiking neural networks with FORCE training
Source: Nat Commun. 2017 Dec 20;8:2208. doi: 10.1038/s41467-017-01827-3 (PMC5738356; doi:10.1038/s41467-017-01827-3)
Supplement: Supplementary file 3 — Description of Additional Supplementary Information [file 41467_2017_1827_MOESM3_ESM.pdf]

Description of Additional Supplementary Files

File Name: Supplementary Movie 1
Description: Two consecutive replays of the songbird supervisor with the neuronal activity in the form of r(t), the synaptically filtered and normalized spike trains plotted as a block matrix on the right.

File Name: Supplementary Movie 2
Description: Replay of the long Ode to Joy clip with the HDTS components in the bottom, and the decoded notes in the top. The network activity is shown on the right in the form of r(t). The video clip is 100 seconds long.

File Name: Supplementary Audio 1
Description: A 1:40 audio clip of the short Ode to Joy spiking network reproducing the song. The decoded network output is used as the envelope for the corresponding note frequency. The wave forms used to generated the song are sinusoidal.
